# Patient Participation in AI for Health Curriculum

**DOI:** 10.2196/69942

**Published:** 2025-09-24

**Authors:** Kirsten Ostherr, Waverly Huang, Ana Park, Tom Punnen, Bhavik Tadigotla, Valencia Robinson, Andrea Downing

**Affiliations:** 1 Medical Humanities Research Institute Rice University Houston, TX United States; 2 Baylor College of Medicine Houston, TX United States; 3 School of Medicine The University of Texas Southwestern Medical Center Dallas, TX United States; 4 Department of Hepatology Baylor College of Medicine Houston, TX United States; 5 The Light Collective Daytona Beach, FL United States; 6 The Light Collective Eugene, OR United States

**Keywords:** artificial intelligence, patient perspectives, experts by experience, premedical education, coproduction of knowledge, ethics

## Abstract

The adoption of artificial intelligence (AI) in health care has outpaced education of the clinical workforce on responsible use of AI in patient care. Although many policy statements advocate safe, ethical, and trustworthy AI, guidance on the use of health AI has rarely included patient perspectives. This gap leaves out a valuable source of information and guidance about what responsible AI means to patients. In this viewpoint coauthored by patients, students, and faculty, we discuss a novel approach to integrating patient perspectives in undergraduate premedical education in the United States that aims to foster an inclusive and patient-centered future of AI in health care.

## Introduction

The rapid uptake of artificial intelligence (AI) in health care has created a need for education of the clinical workforce on responsible, patient-centered use of AI [1,2]. Premedical college students, as the first generation of ChatGPT natives, are in a unique position to engage with innovative pedagogical approaches to health AI as foundational training for their future careers in health care [3,4]. As members of the National Academy of Medicine’s Vital Directions for Health and Health Care: Priorities for 2025 initiative recently observed, “In the same way that training programs for physicians and allied health professionals require prerequisites of study in biology, chemistry, statistics, and anatomy, basic knowledge of AI and its applications is needed for all health care personnel” [5]. We designed an undergraduate course for premedical college students called “Responsible AI for Health” to begin to address this gap. Along with guest lecturers from health care and technology companies, the course included a patient advocate (AD) as an invited speaker. The visit sparked mutual recognition of a unique opportunity to bring our resources together and advance the work of patient advocacy while enhancing the education of the premedical college students. We developed a collaboration linking a subset of premedical students (WH, AP, TP, BT) and the faculty advisor (KO) with a larger group of patient advocates called The Light Collective (AD, VR) to support their work on the “Patient AI Rights Initiative” [6]. In this viewpoint coauthored by patients, students, and faculty, we discuss a novel approach to integrating patient perspectives on AI in undergraduate premedical education in the United States. By partnering with patients as coproducers of knowledge on health technology design and use [7,8], we pilot-tested a method for fostering a workforce trained in ethical, trustworthy, and patient-centered approaches to health AI [9,10]. This small-scale proof of concept may serve as a useful model for educators at all levels to consider integrating patient perspectives on AI in their teaching.

## Patients as Coproducers of Knowledge: The Light Collective

The movement toward integrating patient perspectives into premedical education began decades ago, as modern medical ethics guidelines shifted away from framing patients as passive learning resources [11] to be studied by clinical experts [12]. In recent years, patients have begun to be recognized as essential partners in the knowledge creation process [13,14]. Starting in the early 2000s, a US-based group called the e-patient scholars working group played a pivotal role in identifying the transformative potential of emerging technologies such as the internet to empower and engage patients in peer-to-peer health care [15,16].

Building on that foundation, a group of patient advocates formed The Light Collective in 2018 [17] in the wake of the Cambridge Analytica data breach that exposed the private information of more than 50 million Facebook users [18]. Researchers and patient advocates recognized that health data may have been harvested and mined as part of the data breach [19]. AD, a founding member of The Light Collective, was a Facebook moderator for a support group of over 10,000 cancer previvors and survivors at the time. AD realized that similar lapses might put the privacy of all online communities at risk and began research on application programming interfaces, browser plugins, and developer tools for groups on Facebook’s platform. This research led to the discovery of a security vulnerability that could potentially expose the sensitive personal data shared by members of all closed groups on Facebook. Through partnerships with cybersecurity experts, other patient activists, and legal experts, The Light Collective brought these complaints about Facebook to the Federal Trade Commission [20]. Despite an unsatisfactory Federal Trade Commission ruling on the case, the advocacy increased awareness among the public, the media, and health care professionals about the unique value of patient perspectives on health data privacy [21].

**Figure 1:**
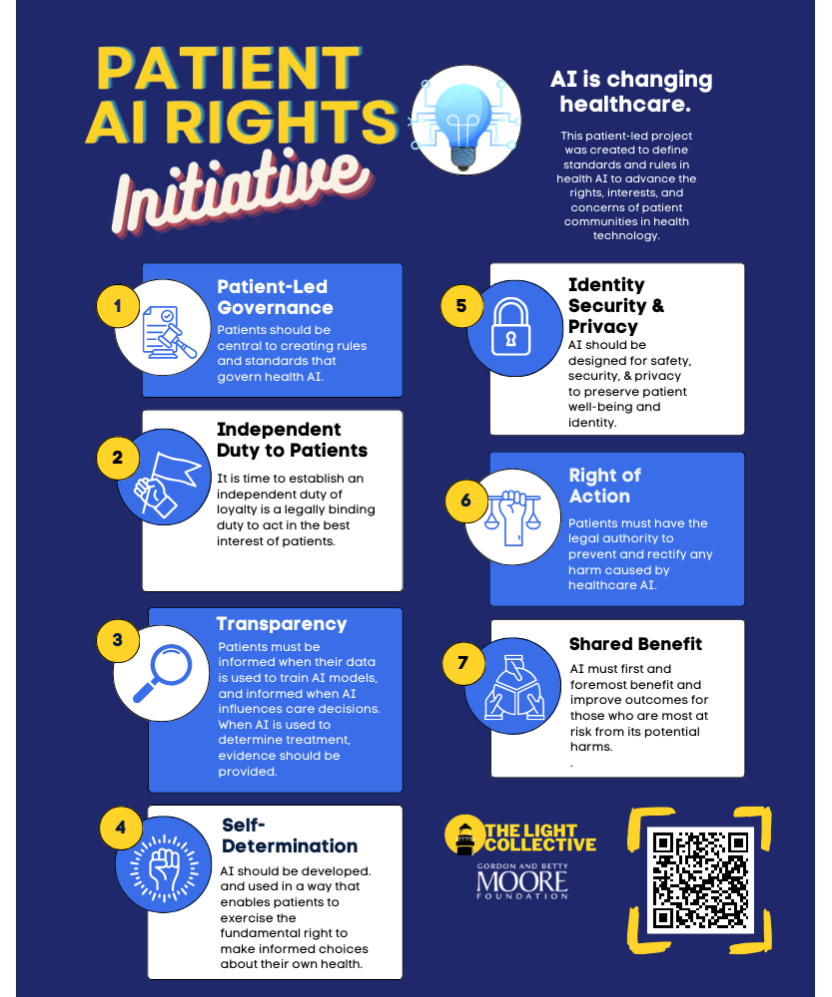
The Light Collective: 7-point summary of the patient AI rights initiative (June 2024; reproduced from The Light Collective [22], with permission from Andrea Downing, co-founder of The Light Collective). AI: artificial intelligence.

In 2023, when GPT-3 was released by OpenAI and rapidly adopted in health care, The Light Collective recognized an unmet need to bring patient community representation into the governance of AI, and the group launched the Patient AI Rights Initiative [6]. The initiative began with patient-led research in partnership with The Light Collective’s diverse and growing coalition of patient advocacy organizations. They invited patient community stakeholders to share their thoughts through public comments and sought input from key stakeholders through private comments and combined these perspectives into a draft of the first patient-led framework for AI rights. Building on that framework, The Light Collective assembled a cohort of 12 participants to further explore patient perspectives on health AI. Each participant was not only an expert by lived experience, but also a person who had made significant efforts to drive change toward integrating patients as partners in clinical research. The cohort was tasked with sharing their knowledge with other community leaders, and biweekly meetings were held from December 2023 to June 2024 as a forum for discussion and debate about emerging priorities from the group. The cohort collaboratively authored a white paper enumerating 7 “Collective AI Rights for Patients,” which was posted online along with an infographic summarizing the results (Figure 1) [22].

## Patients as Partners in the Premedical Classroom: Proof of Concept

In fall 2023, a group of approximately 40 undergraduate college students were enrolled in one of the first humanities-based undergraduate classes in the United States on “Responsible AI for Health” [23], taught at Rice University in Houston, Texas. The 14-week course considered the social, cultural, and ethical issues related to the development and application of AI for health and explored the ways that technology can have unintended consequences that impact individuals and society and may increase existing health disparities. The instructor (KO) had been in conversation with members of The Light Collective dating back to the Cambridge Analytica data breach and invited a member (AD) to give a guest lecture in the class, drawing on their years of experience identifying risks and opportunities related to patient health data sharing. AD presented The Light Collective’s Patient AI Rights Initiative and discussed the 7 principles outlined in that document, focused on centering patient interests as a primary objective of health AI governance. The students who were in the class will enter medical training as ChatGPT natives, having used AI throughout their undergraduate premedical education. In their feedback on AD’s lecture, the students commented that they found the patient perspective compelling and unique. In particular, students noted the contrast with the other perspectives presented through the class readings, which discussed health AI as a technical, ethical, or regulatory challenge, but not as a high-stakes personal opportunity or threat, as it was for the patient advocates.

In a follow-up to the class visit, the faculty member (KO) and advocate (AD) recognized a unique, mutual opportunity to advance the Patient AI Rights Initiative while also advancing the education of the premedical college students. The Light Collective wanted to document and disseminate the insights gained through the initiative; to do so, they partnered with the instructor and 4 premedical undergraduate student research assistants (WH, AP, TP, and BT) with support from the Rice University Medical Humanities Research Institute. The team undertook a structured process of recording and publicizing the narratives emerging from the patient cohort’s learnings, working closely with 2 members of The Light Collective (AD and VR) to collaboratively create a set of video interviews showcasing the results of the Patient AI Rights Initiative [6]. This project brought together the principle of patients as partners with a coproduction of knowledge pedagogy. By participating in this process, the premedical student team learned how to engage in the cocreation of knowledge through a nonhierarchical team structure and methodology, where the questions, priorities, and output were guided by the patient-led cohort and not the academic researchers [24]. In contrast to the more hierarchical model of scientific laboratory work that was familiar to the students, this coproduction model promoted equal partnership between students, patients, and faculty, leading to novel insights that were captured in the students’ reflections on the experience, including the following observations.

…I learned that patients, as the ultimate end users, not only provide invaluable perspectives and innovative ideas often overlooked by health care professionals and technologists but also show a strong commitment to putting in the maximum effort to make these tools as effective as possible, further emphasizing the critical need for their collaboration in developing AI-integrated health care technologies.

WH

…Interviews with advocates revealed the unique stories and backgrounds of patients that were often difficult to hear in typical health care facilities, underscoring the accountability our health care system should feel in bringing the narratives of each individual patient into medicine.

AP

…By truly listening to patients, we can ensure that health care becomes more responsive, inclusive, and better aligned with the real needs of those it serves.

BT

…As a researcher in the field of health AI, I am reminded by this work to consider qualitative, personal insights as data points that inform more humane technology solutions.

TP

The impact of coproduction with patients as teaching partners was evident in the experiences of the premedical student research assistants involved in this project (WH, AP, TP, and BT), who reported that partnership with The Light Collective profoundly shaped their understanding of the critical role that patient collaboration can play in the development of responsible health AI. The students noted that patients, as the ultimate end users, bring perspectives and innovative ideas that health care professionals and technologists might not consider. Several students found that The Light Collective members’ commitment to ensuring that AI tools are as effective and patient-centered as possible further demonstrated the benefits of involving patients in every stage of the development process. Finally, The Light Collective’s interviews with their diverse nationwide community of patient advocates revealed to the students that an understanding of individuals’ unique lived experiences is often missing in traditional health care settings, highlighting the need to treat these qualitative insights with the same importance as clinical data. Through this partnership, the students voiced their appreciation of the patient-led team’s involvement in shaping learning outcomes and capturing the voices of the community from a position of equity. The resulting output was a set of video interviews cocreated with The Light Collective cohort members discussing their views on health AI in their own words, which the students had reviewed, transcribed, and edited in full-length interview format and in short highlight reels. These videos are openly accessible through The Light Collective’s Vimeo page [6].

## Conclusion

The small-scale project we describe here took place in the US context of evolving debates about the role of patient perspectives in shaping policies related to health AI from tool design and piloting to education of the health care workforce. The Association of American Medical Colleges [25] and the American Medical Association [26], as well as numerous medical specialty organizations, have active working groups on the integration of AI into health profession education. These organizations provide resources to help their member institutions develop AI curricula, offer online courses on health AI, and convene virtual communities to discuss AI implementation. Although the American Medical Association acknowledges the importance of transparency regarding AI use in patient care, a shift toward a more patient-centered educational framework is crucial for adequately addressing these concerns within the medical field. The Association of American Medical Colleges’ AI in Academic Medicine series only mentions patients in the context of patient care or patient records [25], not as sources of insight and expertise. An opportunity gap arises by not including patient perspectives to enhance the learnings and skills of clinicians. For example, patients could highlight how AI might contribute to patient self-care, information-seeking, peer-to-peer networking, trust in health care providers and organizations, care interactions, and understanding of cultural contributors to communication.

Although we have described the benefits of integrating patient perspectives into premedical college student education, several challenges to integrating patients into AI medical education are worth noting. Some cultural attitudes within health care may lead to resistance based on differing levels of formally credentialled expertise. A coproduction model in which patients are partnered with clinicians may facilitate the cultural adjustments. Legal concerns related to liability, privacy, and confidentiality of patient data may arise. Explicit instruction in regulatory guidelines will be necessary to address any legal concerns. Logistical issues around already constrained medical schedules may arise. Scheduling issues may be overcome in part by adapting existing trainings to incorporate patient partners, rather than adding new, separate content that would require additional instructional time. Moreover, the opportunity to initiate this educational process at the premedical level warrants further exploration. Addressing these challenges will require thoughtful deliberation and planning to enable medical educators to find feasible methods for integrating patient perspectives.

This viewpoint presents a novel partnership between patient advocates and premedical college students that created a unique opportunity for the coproduction of knowledge about health AI. Based on our experiences, we recommend the integration of patients as partners in teaching as a timely and necessary evolution of the role of patients as research partners. We suggest that the framework presented by The Light Collective can be considered in dialogue with patient advisory committees that already exist at many hospitals and academic health centers to identify the concerns and priorities of local patient populations related to the use of AI in their health care. Nationwide efforts to develop new curricula on health AI have an opportunity to integrate patient perspectives by partnering with organizations like The Light Collective and learning from their work, to ensure the development of safe, trustworthy, and patient-centered AI for health.

## Abbreviations

AI: artificial intelligence
